# Supplementary material for: Crystal violet structural analogues identified by in silico drug repositioning present anti-Trypanosoma cruzi activity through inhibition of proline transporter TcAAAP069
Source: PLoS Negl Trop Dis. 2020 Jan 21;14(1):e0007481. doi: 10.1371/journal.pntd.0007481 (PMC6994103; doi:10.1371/journal.pntd.0007481)
Supplement: S1 Fig — Kinetic analysis of proline uptake was performed using different concentrations of proline (from 0 to 10 mM) in the presence (+ CV) or absence of CV 5 μM (Control). The Lineweaver-Burk plot is showed as an inset. The data is expressed as the mean ± standard deviation and corresponds to three independent experiments. Data were fitted to the Michaelis-Menten equation using GraphPad Prism 6 software. (DOCX) [file pntd.0007481.s001.docx]

**S1 Fig**

**
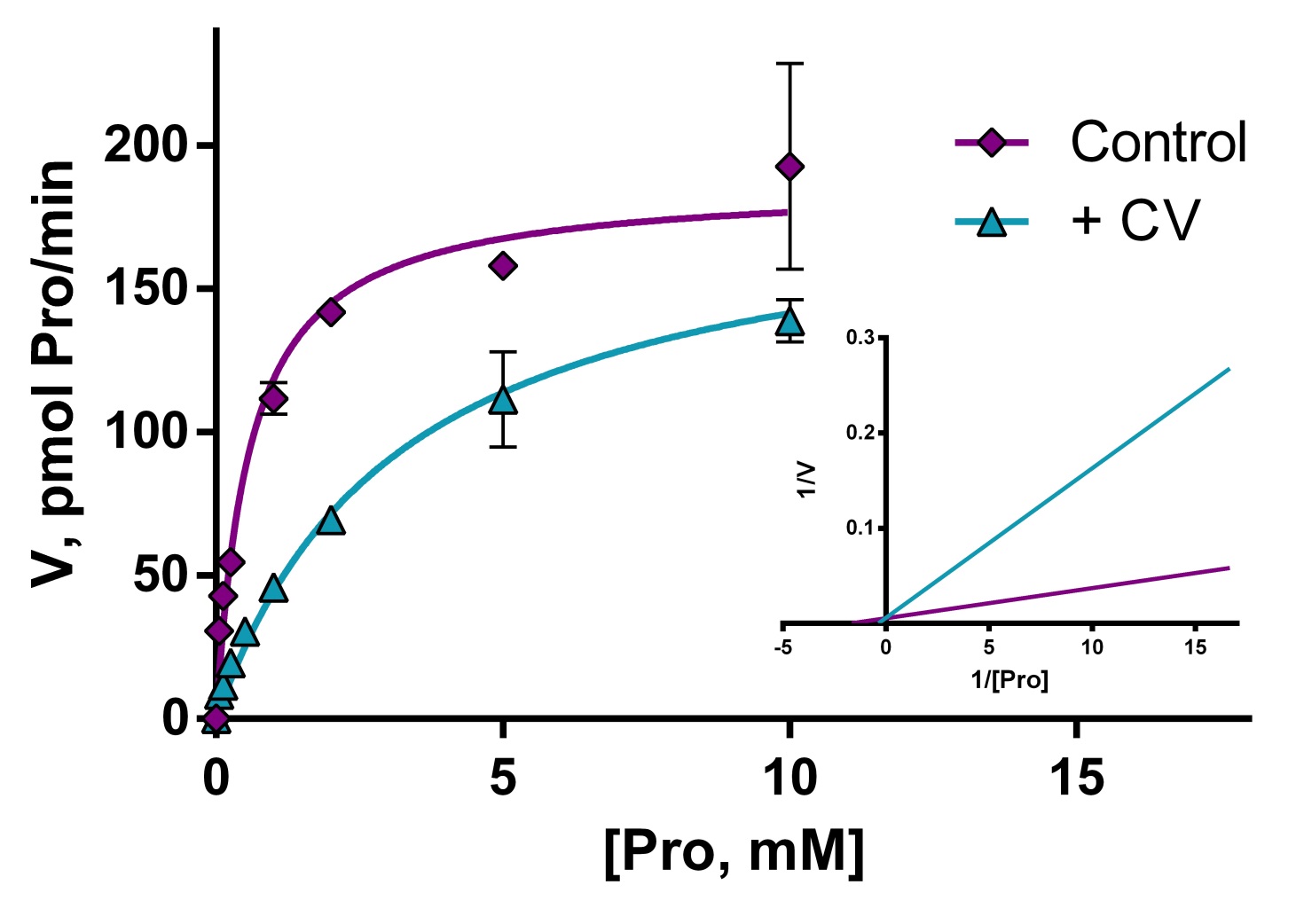
**

**Effect of crystal violet in kinetic parameters of proline uptake**. Kinetic analysis of proline uptake was performed using different concentrations of proline (from 0 to 10 mM) in the presence (+ CV) or absence of CV 5 µM (Control). The Lineweaver-Burk plot is showed as an inset. The data is expressed as the mean ± standard deviation and corresponds to three independent experiments. Data were fitted to the Michaelis-Menten equation using GraphPad Prism 6 software.
