# Supplementary material for: Crystal violet structural analogues identified by in silico drug repositioning present anti-Trypanosoma cruzi activity through inhibition of proline transporter TcAAAP069
Source: PLoS Negl Trop Dis. 2020 Jan 21;14(1):e0007481. doi: 10.1371/journal.pntd.0007481 (PMC6994103; doi:10.1371/journal.pntd.0007481)
Supplement: S2 Fig — The assays were performed in presence (+ CV) or absence of CV 10 μM (Control). a) Proline uptake was measured in regular PBS or PBS containing only K+ (Na+ free buffer) or Na+ (K+ free buffer). b) Proline uptake was measured in citrate buffer at pHs 5.0, 5.5, 6.0 and 6.5. Statistical analysis was performed considering the transport at pH 5.0 as 100% for each treatment (Control and + CV) and then the curves were adjusted to a linear regression in order to compare the slopes. The data is expressed as the mean ± standard deviation and corresponds to three independent experiments. (DOCX) [file pntd.0007481.s002.docx]

**S2 Fig**

**
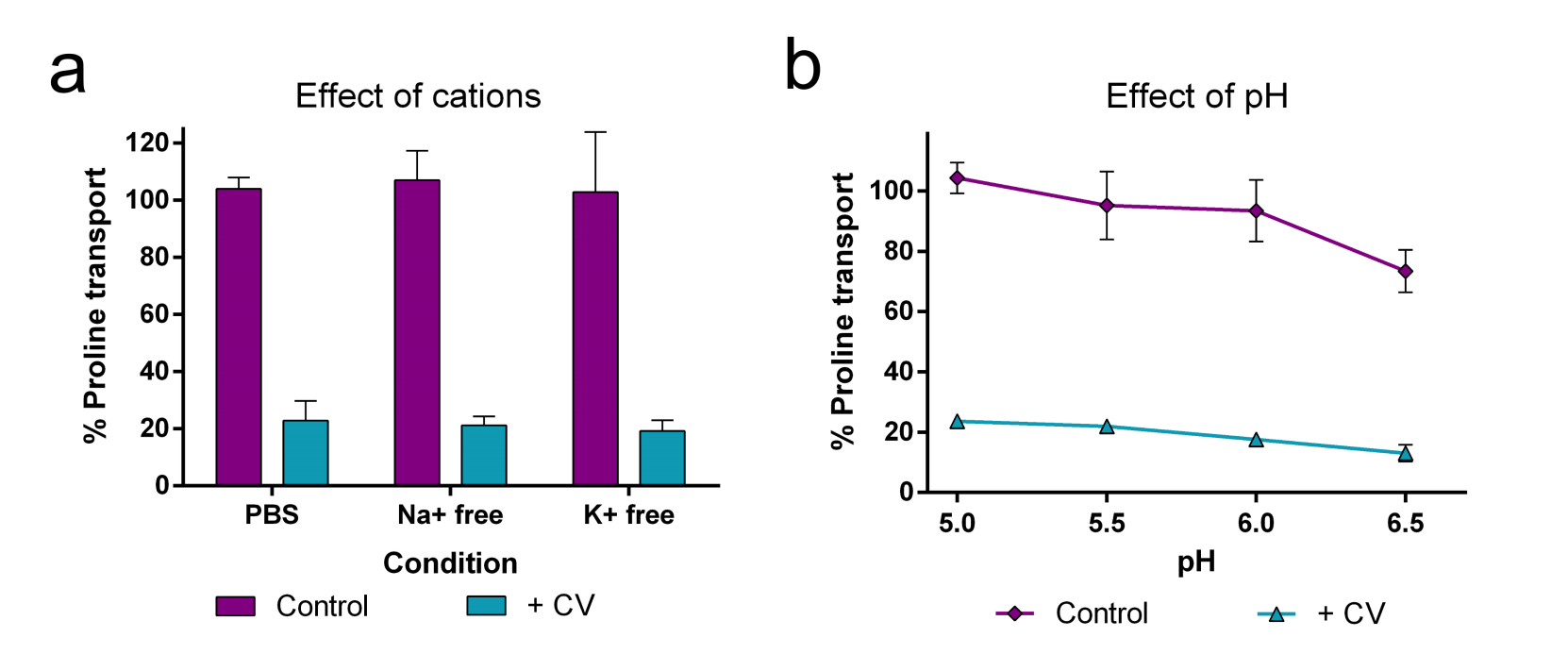
**

**Effect of extracellular ions in CV proline uptake inhibition**. The assays were performed in presence (+ CV) or absence of CV 10 µM (Control). a) Proline uptake was measured in regular PBS or PBS containing only K^+^ (Na^+^ free buffer) or Na^+^ (K^+^ free buffer). b) Proline uptake was measured in citrate buffer at pHs 5.0, 5.5, 6.0 and 6.5. Statistical analysis was performed considering the transport at pH 5.0 as 100% for each treatment (Control and + CV) and then the curves were adjusted to a linear regression in order to compare the slopes. The data is expressed as the mean ± standard deviation and corresponds to three independent experiments.
