# Supplementary material for: Crystal violet structural analogues identified by in silico drug repositioning present anti-Trypanosoma cruzi activity through inhibition of proline transporter TcAAAP069
Source: PLoS Negl Trop Dis. 2020 Jan 21;14(1):e0007481. doi: 10.1371/journal.pntd.0007481 (PMC6994103; doi:10.1371/journal.pntd.0007481)
Supplement: S3 Fig — The LigandScout software was used to identify the common chemical features between crystal violet (CV) and its chemical analogues. This algorithm performs feature-based structure alignments where the similarities are calculated as the number of matched feature pairs (MFP). (a) In order to perform the structure comparisons, the 8 CV features were set as references. (b) The LigandScout similarity score, the number of MFP obtained for each compound is listed and the root mean square (RMS) of their positions is included. (c) The features shared between CV and each compound are shown and the structure alignments with the van der Waals surfaces are schematized. N/A, not available. AR, aromatic ring (purple circles). H, hydrophobic area (yellow remarks). PI, positive ionizable atom (purple lines). (DOCX) [file pntd.0007481.s003.docx]

**S3 Fig**


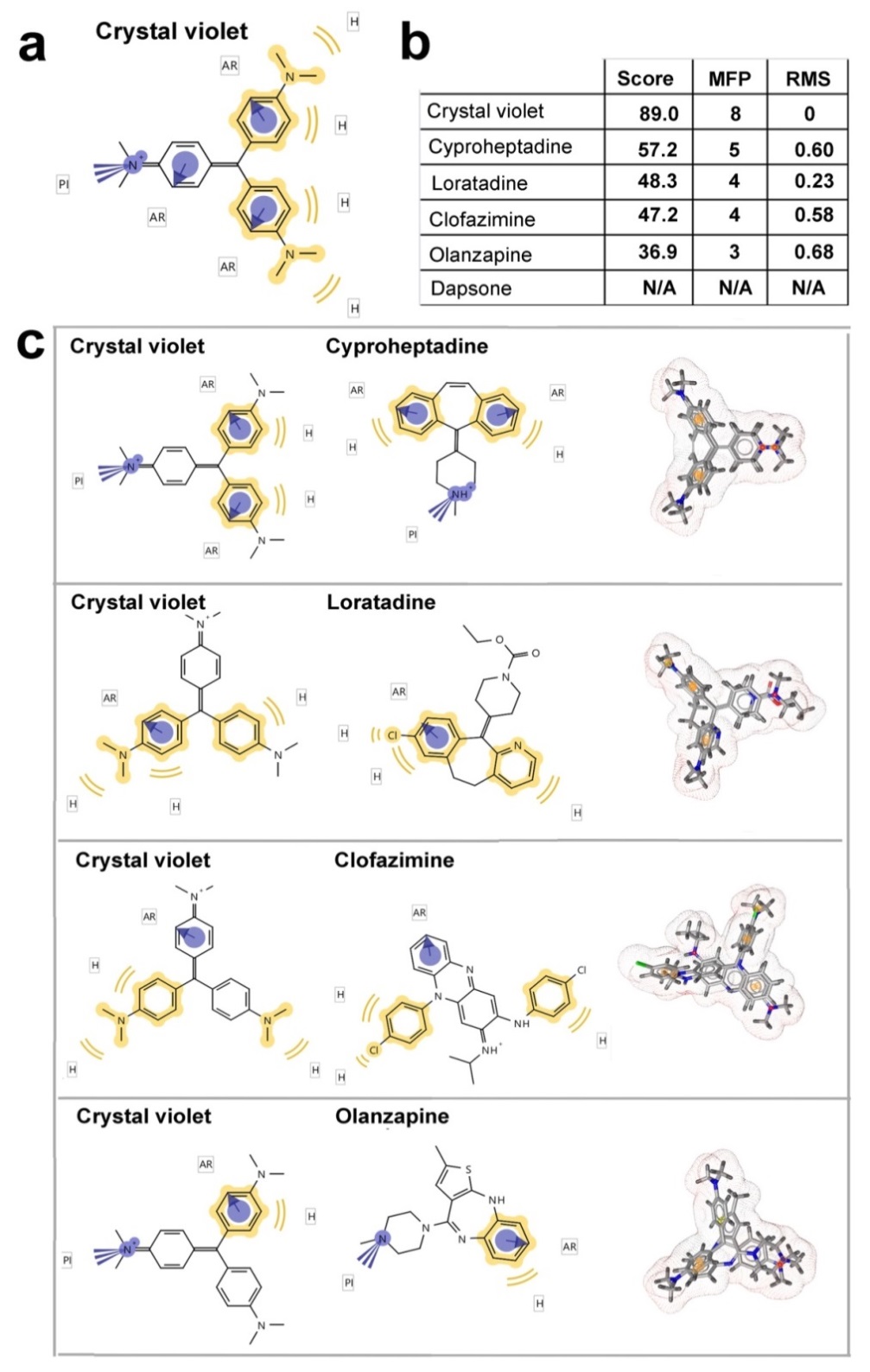


**Chemical features shared between crystal violet and the selected compounds**. The LigandScout software was used to identify the common chemical features between crystal violet (CV) and its chemical analogues. This algorithm performs feature-based structure alignments where the similarities are calculated as the number of matched feature pairs (MFP). (a) In order to perform the structure comparisons, the 8 CV features were set as references. (b) The LigandScout similarity score, the number of MFP obtained for each compound is listed and the root mean square (RMS) of their positions is included. (c) The features shared between CV and each compound are shown and the structure alignments with the van der Waals surfaces are schematized. N/A, not available. AR, aromatic ring (purple circles). H, hydrophobic area (yellow remarks). PI, positive ionizable atom (purple lines).
