# Supplementary material for: Crystal violet structural analogues identified by in silico drug repositioning present anti-Trypanosoma cruzi activity through inhibition of proline transporter TcAAAP069
Source: PLoS Negl Trop Dis. 2020 Jan 21;14(1):e0007481. doi: 10.1371/journal.pntd.0007481 (PMC6994103; doi:10.1371/journal.pntd.0007481)
Supplement: S4 Fig — I. Predicted transmembrane spans of proline permease TcAAAP069. Transmembrane spans were predicted with TOPCONS software (http://topcons.cbr.su.se) and are numbered from 1 to 11. II. Predicted poses by molecular docking of the crystal violet structural analogues and the proline permease TcAAAP069. Residues corresponding to the PRO and CV sites in TcAAAP069 are indicated in green and violet, respectively. Detail of the TcAAAP069 residues predicted to interact with (a) clofazimine, (b) loratadine, (c) cyproheptadine and (d) olanzapine. (DOCX) [file pntd.0007481.s004.docx]

**S4 Fig**

**
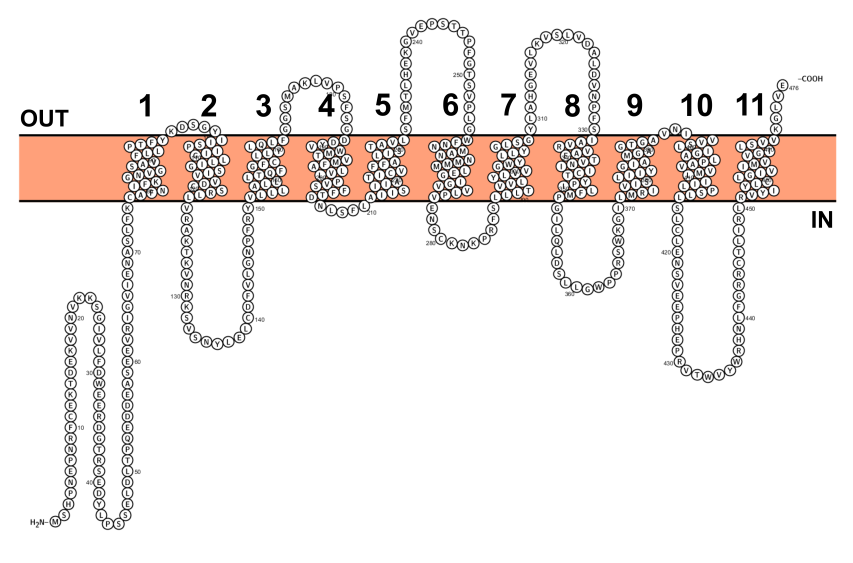
**

**I.**

**
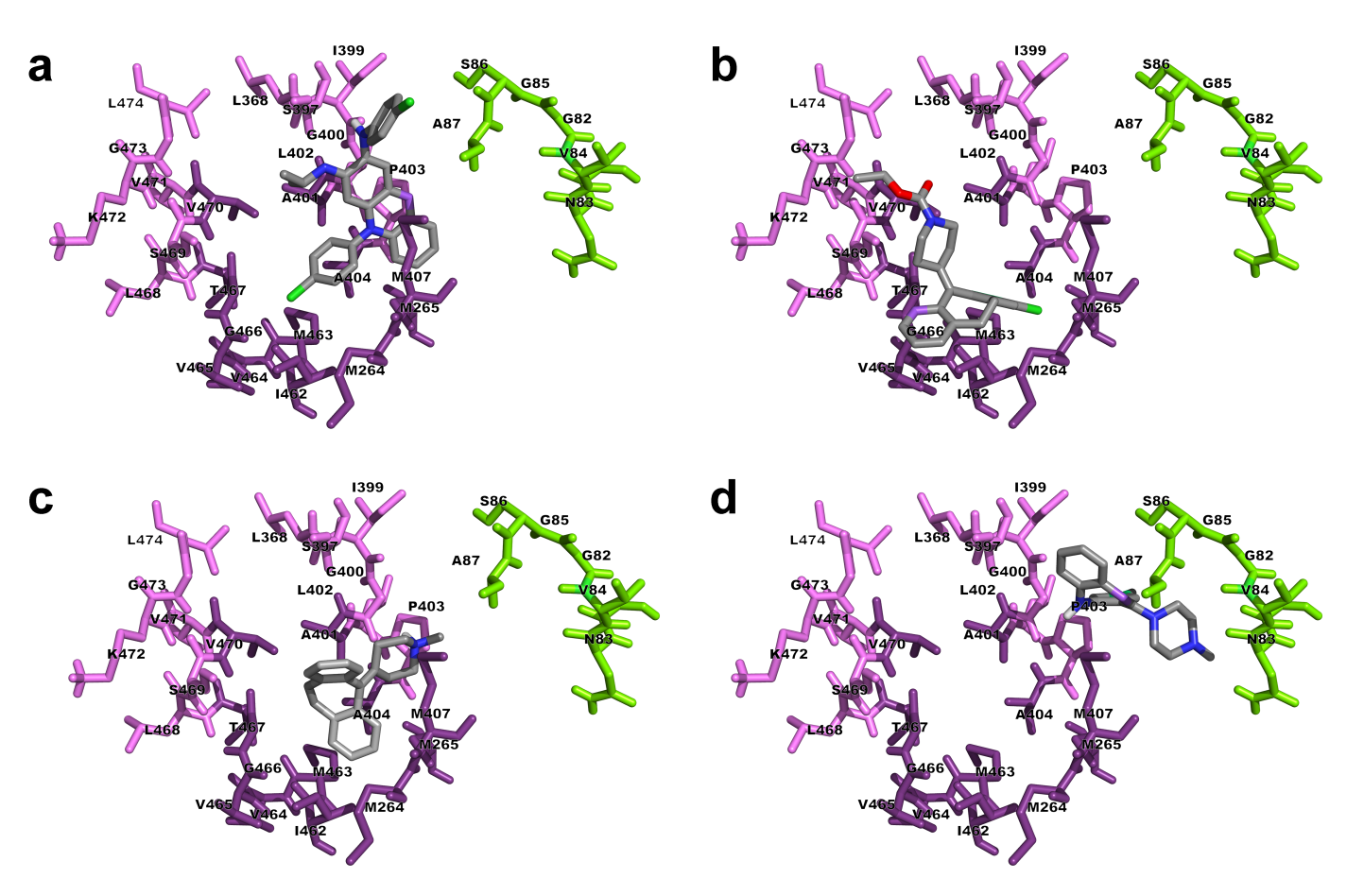
**

**II.**

**I. Predicted transmembrane spans of proline permease TcAAAP069.** Transmembrane spans were predicted with TOPCONS software (<http://topcons.cbr.su.se>) and are numbered from 1 to 11. **II. Predicted poses by molecular docking of the crystal violet structural analogues and the proline permease TcAAAP069.** Residues corresponding to the PRO and CV sites in TcAAAP069 are indicated in green and violet, respectively. Detail of the TcAAAP069 residues predicted to interact with (a) clofazimine, (b) loratadine, (c) cyproheptadine and (d) olanzapine.
