# Supplementary material for: Crystal violet structural analogues identified by in silico drug repositioning present anti-Trypanosoma cruzi activity through inhibition of proline transporter TcAAAP069
Source: PLoS Negl Trop Dis. 2020 Jan 21;14(1):e0007481. doi: 10.1371/journal.pntd.0007481 (PMC6994103; doi:10.1371/journal.pntd.0007481)
Supplement: S5 Fig — The crystal violet analogues and dapsone were evaluated as potential proline transport inhibitors at two concentrations, 25 and 100 μM. Control, no treatment. The data is expressed as the mean ± standard deviation and corresponds to three independent experiments. DPS, dapsone. LTD, loratadine. CPH, cyproheptadine. OLZ, olanzapine. CFZ, clofazimine. *, p< 0.05; ***, p< 0.001; ****, p<0.0001. ns, not significant. (DOCX) [file pntd.0007481.s005.docx]

**S5 Fig**

**
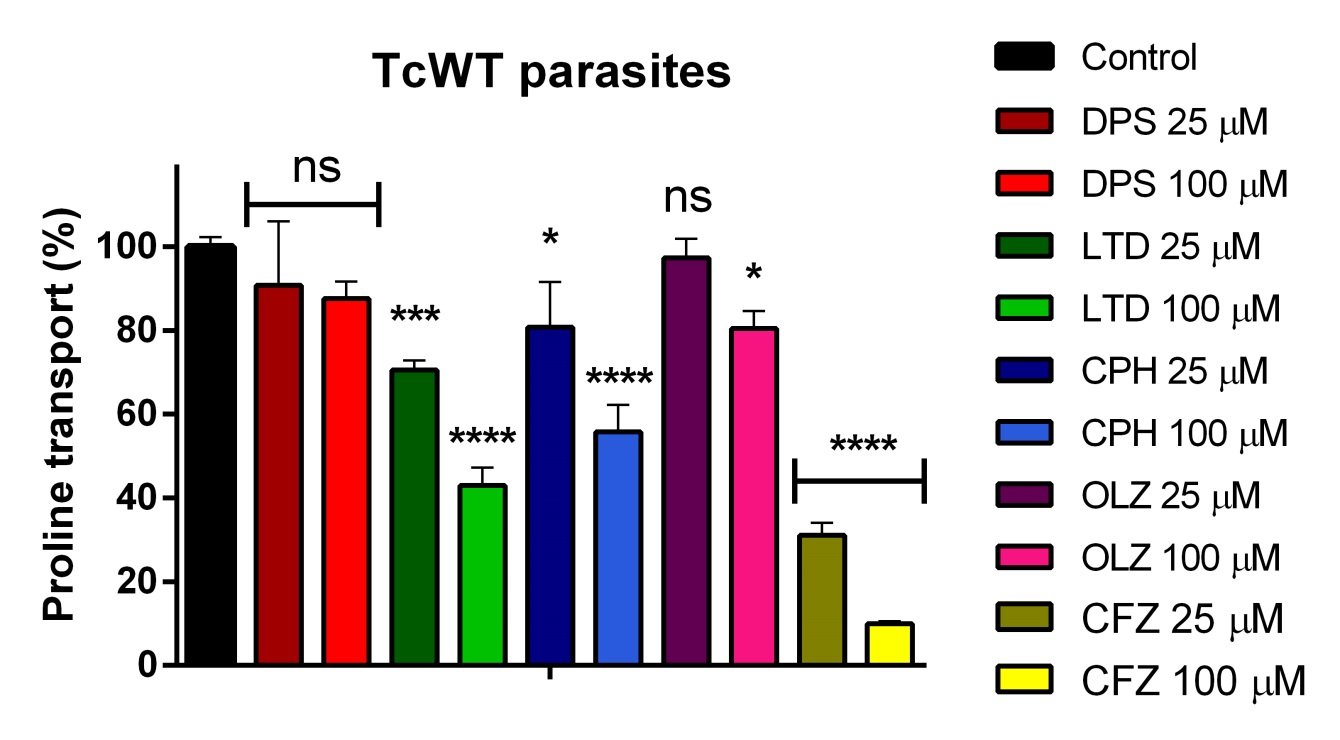
**

**Inhibition of proline transport by crystal violet chemical analogues in wild type parasites (TcWT).** The crystal violet analogues and dapsone were evaluated as potential proline transport inhibitors at two concentrations, 25 and 100 µM. Control, no treatment. The data is expressed as the mean ± standard deviation and corresponds to three independent experiments. DPS, dapsone. LTD, loratadine. CPH, cyproheptadine. OLZ, olanzapine. CFZ, clofazimine. *, p< 0.05; ***, p< 0.001; ****, p<0.0001. ns, not significant.
