# Supplementary material for: Crystal violet structural analogues identified by in silico drug repositioning present anti-Trypanosoma cruzi activity through inhibition of proline transporter TcAAAP069
Source: PLoS Negl Trop Dis. 2020 Jan 21;14(1):e0007481. doi: 10.1371/journal.pntd.0007481 (PMC6994103; doi:10.1371/journal.pntd.0007481)
Supplement: S6 Fig — Trypanocidal effect of CV structural analogues in (a) epimastigotes, (b) trypomastigotes and (c) amastigotes of T. cruzi Y strain. The concentrations required to inhibit 50% of parasite growth or parasite survival were calculated for the four CV chemical analogues. The data is expressed as the mean ± standard deviation and corresponds to three independent experiments. BZL, benznidazole. CV, crystal violet. LTD, loratadine. CPH, cyproheptadine. OLZ, olanzapine. CFZ, clofazimine. N/A, not available. (DOCX) [file pntd.0007481.s006.docx]

**S6 Fig**

**
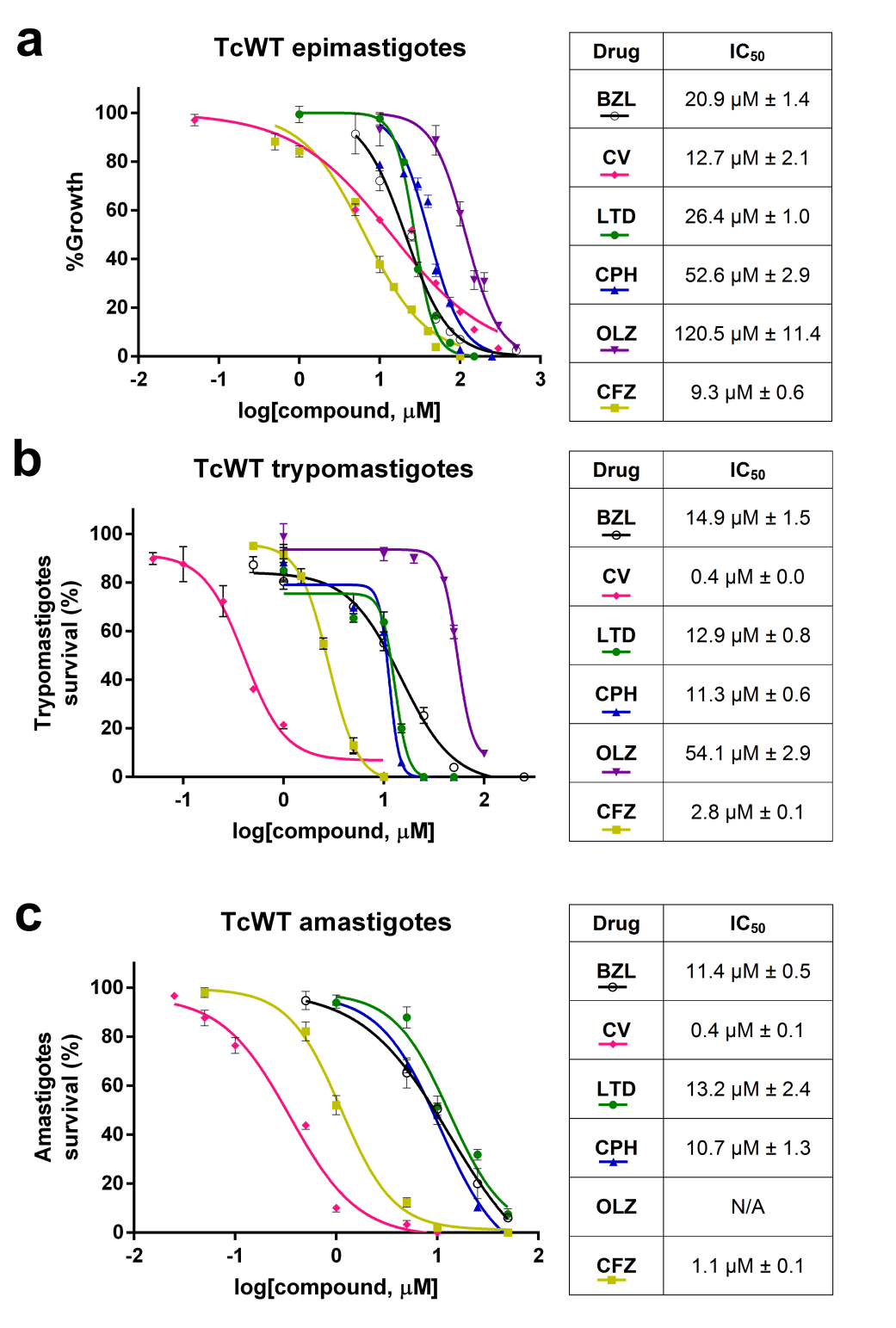
**

**Trypanocidal effect of CV structural analogues in (a) epimastigotes, (b) trypomastigotes and (c) amastigotes of *T. cruzi* Y strain.** The concentrations required to inhibit 50% of parasite growth or parasite survival were calculated for the four CV chemical analogues. The data is expressed as the mean ± standard deviation and corresponds to three independent experiments. BZL, benznidazole. CV, crystal violet. LTD, loratadine. CPH, cyproheptadine. OLZ, olanzapine. CFZ, clofazimine. N/A, not available.
