# Supplementary material for: Crystal violet structural analogues identified by in silico drug repositioning present anti-Trypanosoma cruzi activity through inhibition of proline transporter TcAAAP069
Source: PLoS Negl Trop Dis. 2020 Jan 21;14(1):e0007481. doi: 10.1371/journal.pntd.0007481 (PMC6994103; doi:10.1371/journal.pntd.0007481)
Supplement: S7 Fig — Trypanocidal effect of CV structural analogues concentrations in epimastigotes of (a) T. cruzi Dm28c and (b) CL Brener strains. The concentrations required to inhibit 50% of parasite growth were calculated for three CV chemical analogues. OLZ was not tested in these strains because of the high IC50s values obtained in trypomastigotes and epimastigotes of the Y strain. The data is expressed as the mean ± standard deviation and corresponds to three independent experiments. BZL, benznidazole. CV, crystal violet. LTD, loratadine. CPH, cyproheptadine. OLZ, olanzapine. CFZ, clofazimine. N/A, not available. (DOCX) [file pntd.0007481.s007.docx]

**S7 Fig**

**
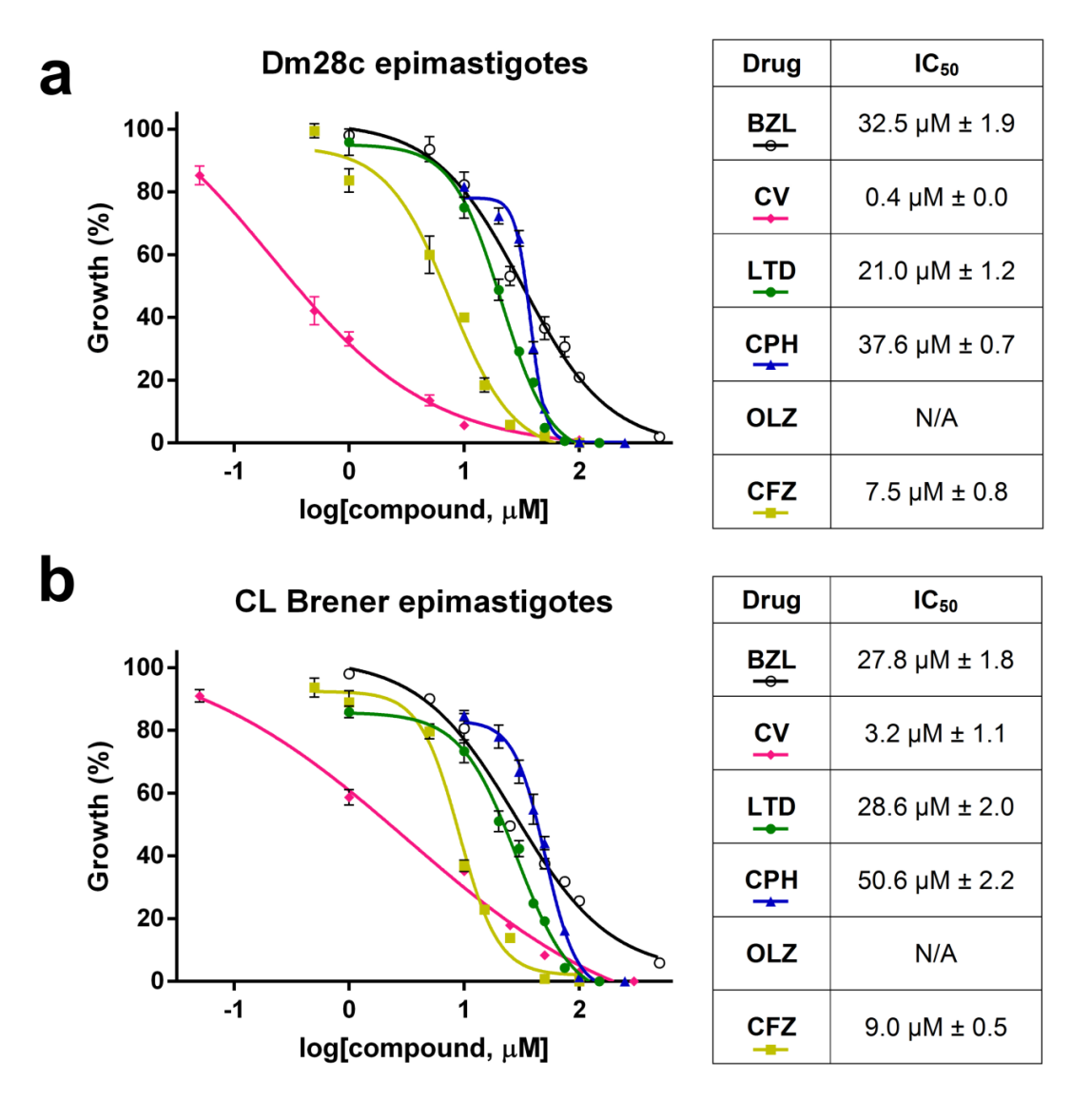
**

**Trypanocidal effect of CV structural analogues concentrations in epimastigotes of (a) *T. cruzi* DM28c and (b) CL Brener strains.** The concentrations required to inhibit 50% of parasite growth were calculated for three CV chemical analogues. OLZ was not tested in these strains because of the high IC_50_s values obtained in trypomastigotes and epimastigotes of the Y strain. The data is expressed as the mean ± standard deviation and corresponds to three independent experiments. BZL, benznidazole. CV, crystal violet. LTD, loratadine. CPH, cyproheptadine. OLZ, olanzapine. CFZ, clofazimine. N/A, not available.
