# Supplementary material for: Crystal violet structural analogues identified by in silico drug repositioning present anti-Trypanosoma cruzi activity through inhibition of proline transporter TcAAAP069
Source: PLoS Negl Trop Dis. 2020 Jan 21;14(1):e0007481. doi: 10.1371/journal.pntd.0007481 (PMC6994103; doi:10.1371/journal.pntd.0007481)
Supplement: S9 Fig — The amastigotes were treated with two concentrations of each compound in order to compare the response of each strain. a) Benznidazole (BZL). b) Crystal violet, (CV). c) Loratadine (LTD). d) Cyproheptadine (CPH). e) Clofazimine (CFZ). The data is expressed as the mean ± standard deviation and corresponds to three independent experiments. *, p<0.05. **, p<0.01. (DOCX) [file pntd.0007481.s009.docx]

**S9 Fig**

**
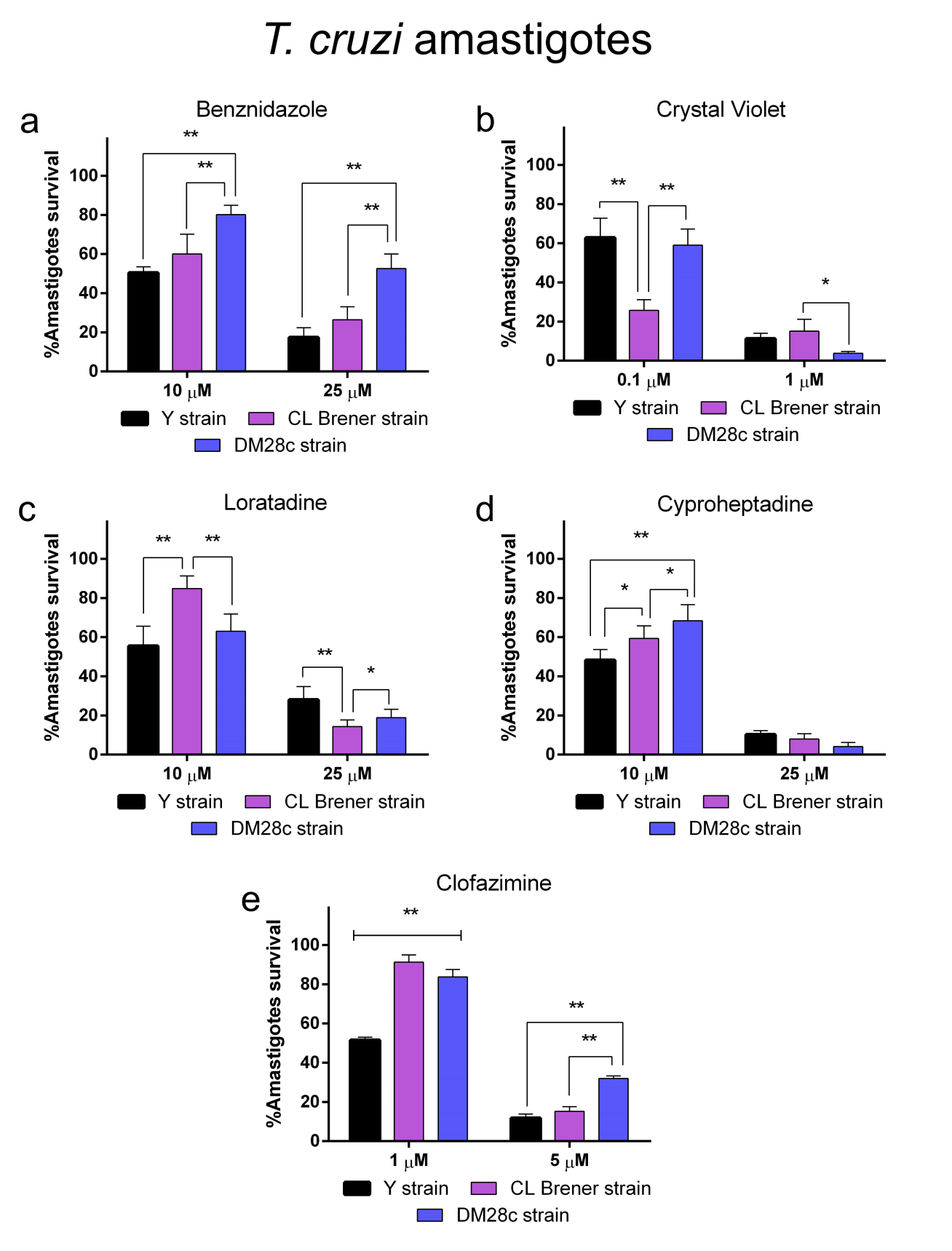
**

**Trypanocidal effect of CV structural analogues in amastigotes of *T. cruzi* DM28c and CL Brener strains.** The amastigotes were treated with two concentrations of each compound in order to compare the response of each strain. a) Benznidazole (BZL). b) Crystal violet, (CV). c) Loratadine (LTD). d) Cyproheptadine (CPH). e) Clofazimine (CFZ). The data is expressed as the mean ± standard deviation and corresponds to three independent experiments. *, p<0.05. **, p<0.01.
