# Supplementary material for: Crystal violet structural analogues identified by in silico drug repositioning present anti-Trypanosoma cruzi activity through inhibition of proline transporter TcAAAP069
Source: PLoS Negl Trop Dis. 2020 Jan 21;14(1):e0007481. doi: 10.1371/journal.pntd.0007481 (PMC6994103; doi:10.1371/journal.pntd.0007481)
Supplement: S10 Fig — (a) Drug combinations between BZL and CV analogues (LTD-CPH-CFZ). Combination index (CI) value for each combination point is presented under the corresponding graded symbol. Graded symbols mean strong synergism (++++, CI between 0.1–0.3), synergism (+++, CI between 0.3–0.7), moderate synergism (++, CI between 0.7–0.85), nearly additive effect (±, CI between 0.9–1.1), and moderate antagonism (- -, CI between 1.2–1.45) [81]. The boxes coloured with light-grey correspond to the combination points where no synergism was observed. (b) Chou-Talalay plot. Representation of CI vs effect (Fa, fraction affected), where CI > 1, CI = 1 (dotted line) and CI < 1 indicate antagonism, additive effect and synergism, respectively. For each data series BZL concentrations increase from left to right (from 0.1 to 20 μM). The data is expressed as the mean ± standard deviation and corresponds to three independent experiments. All calculations were performed with CompuSyn software. LTD, loratadine. CPH, cyproheptadine. CFZ, clofazimine. BZL, benznidazole. IC50 LTD = 25 μM. IC50 CPH = 50 μM. IC50 CFZ = 10 μM. LTD-CPH-CFZ, combination of the three crystal violet analogues as a single drug. 1/2 IC50, refers to the sum of half of each IC50, 12.5 μM + 25 μM + 5 μM = 42.5 μM. 1/5 IC50, 5 μM + 10 μM + 2 μM = 17 μM. 1/10 IC50, 2.5 μM + 5 μM + 1 μM = 8.5 μM. 1/25 IC50, 1 μM + 2 μM + 0.4 μM = 3.4 μM. (DOCX) [file pntd.0007481.s010.docx]

**S10 Fig**

**
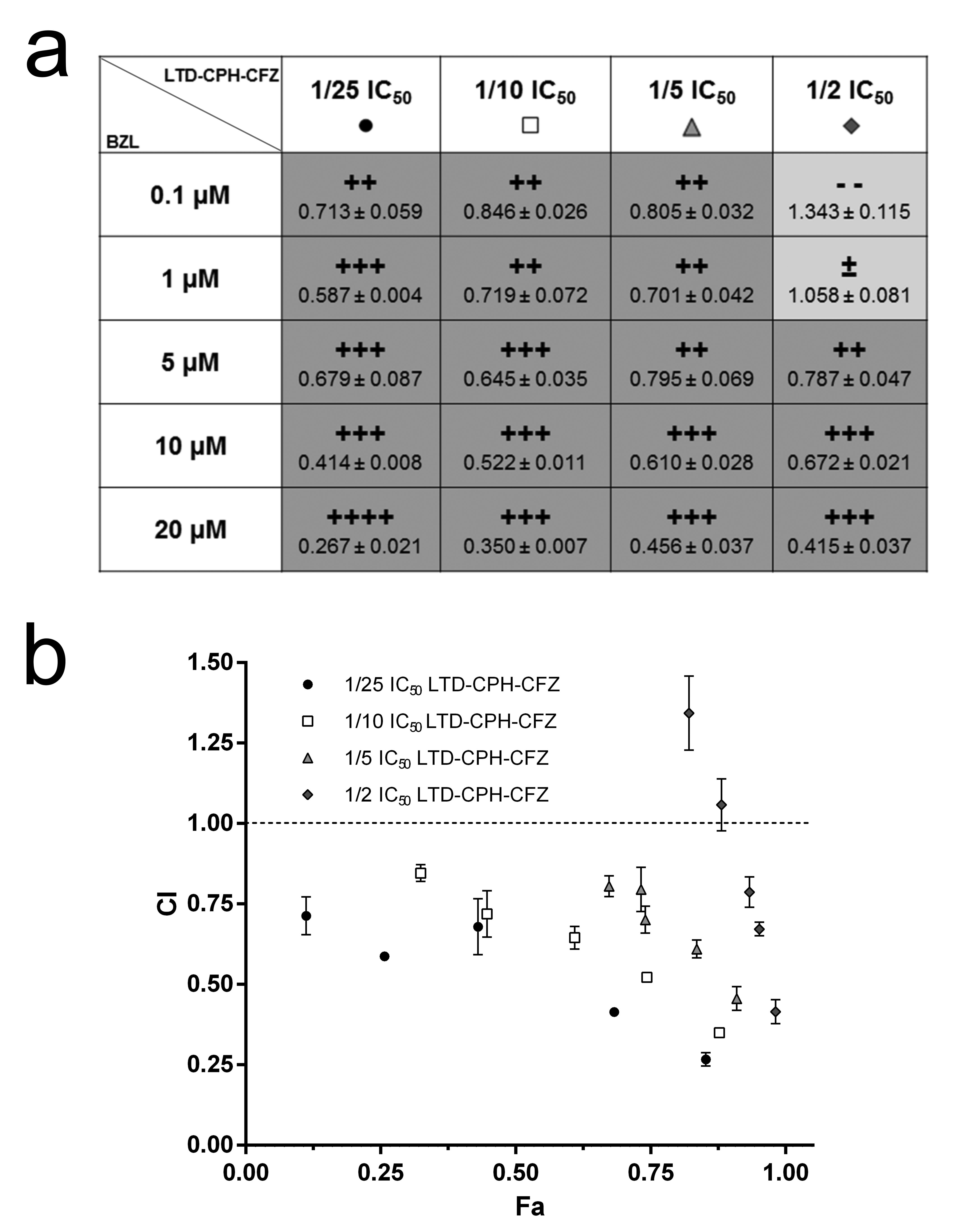
**

**Synergism between benznidazole and the combination of the crystal violet analogues in *T. cruzi* epimastigotes.** (a) Drug combinations between BZL and CV analogues (LTD-CPH-CFZ). Combination index (CI) value for each combination point is presented under the corresponding graded symbol. Graded symbols mean strong synergism (++++, CI between 0.1-0.3), synergism (+++, CI between 0.3-0.7), moderate synergism (++, CI between 0.7-0.85), nearly additive effect (±, CI between 0.9-1.1), and moderate antagonism (- -, CI between 1.2-1.45) [81]. The boxes coloured with light-grey correspond to the combination points where no synergism was observed. (b) Chou-Talalay plot. Representation of CI vs effect (Fa, fraction affected), where CI > 1, CI = 1 (dotted line) and CI < 1 indicate antagonism, additive effect and synergism, respectively. For each data series BZL concentrations increase from left to right (from 0.1 to 20 µM). The data is expressed as the mean ± standard deviation and corresponds to three independent experiments. All calculations were performed with CompuSyn software. LTD, loratadine. CPH, cyproheptadine. CFZ, clofazimine. BZL, benznidazole. IC_50_ LTD = 25 µM. IC_50_ CPH = 50 µM. IC_50_ CFZ = 10 µM. LTD-CPH-CFZ, combination of the three crystal violet analogues as a single drug. 1/2 IC_50_, refers to the sum of half of each IC_50_, 12.5 µM + 25 µM + 5 µM = 42.5 µM. 1/5 IC_50_, 5 µM + 10 µM + 2 µM = 17 µM. 1/10 IC_50_, 2.5 µM + 5 µM + 1 µM = 8.5 µM. 1/25 IC_50_, 1 µM + 2 µM + 0.4 µM = 3.4 µM.
