# Supplementary material for: Crystal violet structural analogues identified by in silico drug repositioning present anti-Trypanosoma cruzi activity through inhibition of proline transporter TcAAAP069
Source: PLoS Negl Trop Dis. 2020 Jan 21;14(1):e0007481. doi: 10.1371/journal.pntd.0007481 (PMC6994103; doi:10.1371/journal.pntd.0007481)
Supplement: S1 Table — (DOCX) [file pntd.0007481.s011.docx]

**S1 Table. Dose-reduction index for multidrug treatment with benznidazole and crystal violet chemical analogues in wild type epimastigotes of *T. cruzi* Y strain.**

| **Fa** | **Dose BZL alone (µM)** | **Dose LTD-CPH-CFZ alone (µM)** | **Dose BZL in combination (µM)** | **Dose LTD-CPH-CFZ in combination (µM)** | **DRI BZL** | **DRI**  **LTD-CPH-CFZ** |
| --- | --- | --- | --- | --- | --- | --- |
| 0.112 | 1.66 ± 0.28 | 5.23 ± 0.39 | 0.10 | 3.40 (1/25 IC_50_) | 16.63 ± 2.78 | 1.54 ± 0.12 |
| 0.324 | 7.37 ± 0.49 | 10.22 ± 0.31 | 0.10 | 8.50 (1/10 IC_50_) | 73.67 ± 4.95 | 1.20 ± 0.04 |
| 0.673 | 37.42 ± 3.25 | 21.22 ± 0.83 | 0.10 | 17.00 (1/5 IC_50_) | 374.17 ± 32.50 | 1.25 ± 0.05 |
| 0.821 | 92.37 ± 17.51 | 31.79 ± 2.72 | 0.10 | 42.50 (1/2 IC_50_) | 923.66 ± 175.14 | 0.75 ± 0.06 |
| 0.257 | 5.12 ± 0.06 | 8.69 ± 0.05 | 1.00 | 3.40 (1/25 IC_50_) | 5.12 ± 0.06 | 2.55 ± 0.01 |
| 0.447 | 13.27 ± 2.61 | 13.29 ± 1.18 | 1.00 | 8.50 (1/10 IC_50_) | 13.27 ± 2.61 | 1.56 ± 0.14 |
| 0.740 | 53.83 ± 6.79 | 24.97 ± 1.42 | 1.00 | 17.00 (1/5 IC_50_) | 53.83 ± 6.79 | 1.47 ± 0.08 |
| 0.881 | 157.01 ± 24.63 | 40.38 ± 2.86 | 1.00 | 42.50 (1/2 IC_50_) | 157.01 ± 24.63 | 0.95 ± 0.07 |
| 0.430 | 12.27 ± 2.01 | 12.84 ± 0.95 | 5.00 | 3.40 (1/25 IC_50_) | 2.45 ± 0.40 | 3.78 ± 0.28 |
| 0.609 | 27.38 ± 2.41 | 18.44 ± 0.73 | 5.00 | 8.50 (1/10 IC_50_) | 5.48 ± 0.48 | 2.17 ± 0.09 |
| 0.732 | 51.57 ± 8.60 | 24.48 ± 1.84 | 5.00 | 17.00 (1/5 IC_50_) | 10.31 ± 1.72 | 1.44 ± 0.11 |
| 0.933 | 315.46 ± 41.27 | 55.28 ± 3.26 | 5.00 | 42.50 (1/2 IC_50_) | 63.09 ± 8.25 | 1.30 ± 0.08 |
| 0.682 | 38.95 ± 1.06 | 21.61 ± 0.26 | 10.00 | 3.40 (1/25 IC_50_) | 3.89 ± 0.11 | 6.36 ± 0.08 |
| 0.743 | 54.45 ± 1.86 | 25.13 ± 0.39 | 10.00 | 8.50 (1/10 IC_50_) | 5.45 ± 0.19 | 2.96 ± 0.05 |
| 0.835 | 101.97 ± 8.68 | 33.30 ± 1.28 | 10.00 | 17.00 (1/5 IC_50_) | 10.20 ± 0.87 | 1.96 ± 0.08 |
| 0.951 | 457.73 ± 30.35 | 65.40 ± 1.95 | 10.00 | 42.50 (1/2 IC_50_) | 45.77 ± 3.03 | 1.54 ± 0.05 |
| 0.852 | 117.55 ± 11.14 | 35.49 ± 1.51 | 20.00 | 3.40 (1/25 IC_50_) | 5.88 ± 0.56 | 10.44 ± 0.45 |
| 0.877 | 148.83 ± 4.56 | 39.48 ± 0.54 | 20.00 | 8.50 (1/10 IC_50_) | 7.44 ± 0.23 | 4.64 ± 0.06 |
| 0.909 | 218.81 ± 31.25 | 46.89 ± 3.02 | 20.00 | 17.00 (1/5 IC_50_) | 10.94 ± 1.56 | 2.76 ± 0.18 |
| 0.981 | 1370.11 ± 265.72 | 106.81 ± 9.36 | 20.00 | 42.50 (1/2 IC_50_) | 68.51 ± 13.29 | 2.51 ± 0.22 |

Fa, fraction affected. DRI, dose-reduction index. Dose alone indicates the concentration of each drug alone needed to achieve the same effect (Fa) as in the combination. BZL, benznidazole. LTD, loratadine. CPH, cyproheptadine. CFZ, clofazimine. IC_50_ LTD = 25 µM. IC_50_ CPH = 50 µM. IC_50_ CFZ = 10 µM. LTD-CPH-CFZ, combination of the three crystal violet analogues as a single drug. 1/2 IC_50_, refers to the sum of half of each IC_50_, 12.5 µM + 25 µM + 5 µM = 42.5 µM. 1/5 IC_50_, 5 µM + 10 µM + 2 µM = 17 µM. 1/10 IC_50_, 2.5 µM + 5 µM + 1 µM = 8.5 µM. 1/25 IC_50_, 1 µM + 2 µM + 0.4 µM = 3.4 µM.
