# Supplementary material for: Crystal violet structural analogues identified by in silico drug repositioning present anti-Trypanosoma cruzi activity through inhibition of proline transporter TcAAAP069
Source: PLoS Negl Trop Dis. 2020 Jan 21;14(1):e0007481. doi: 10.1371/journal.pntd.0007481 (PMC6994103; doi:10.1371/journal.pntd.0007481)
Supplement: S3 Table — (DOCX) [file pntd.0007481.s013.docx]

**S3 Table. Effect of multidrug treatment with benznidazole and crystal violet chemical analogues in VERO cells.**

|  |  | **Dose LTD-CPH-CFZ in combination** | | | | | |
| --- | --- | --- | --- | --- | --- | --- | --- |
|  |  | **0 µM** | **6.0 µM**  **(1/5 IC50)** | **7.5 µM**  **(1/4 IC_50_)** | **10.0 µM**  **(1/3 IC_50_)** | **15.0 µM**  **(1/2 IC_50_)** | **30.0 µM**  **(1/1 IC_50_)** |
| **Dose BZL** | **0** **µM** | 100.0% ± 3.2 | 95.7% ± 3.3 | 95.5% ± 0.4 | 95.7% ± 4.0 | 95.5% ± 0.6 | 83.9% ± 0.2 |
|  | **5.0 µM** | 96.6% ± 3.5 | 90.6% ± 2.1 | 90.0% ± 5.7 | 92.8% ± 1.3 | 88.2% ± 7.1 | 84.3% ± 2.0 |
|  | **10.0 µM** | 97.5% ± 2.1 | 89.6% ± 2.1 | 91.9% ± 0.6 | 88.8% ± 5.9 | 92.9% ± 1.6 | 83.0% ± 2.0 |
|  | **50.0 µM** | 95.7% ± 1.7 | 94.0% ± 2.3 | 93.3% ± 1.4 | 92.5% ± 3.8 | 88.9% ± 0.4 | 79.8% ± 0.4 |
|  | **100.0 µM** | 92.9% ± 6.5 | 91.5% ± 3.0 | 88.0% ± 2.1 | 89.4% ± 4.9 | 87.5% ± 2.9 | 76.7% ± 2.9 |

The viability of the VERO cells is expressed as percentage of untreated cells. Light-grey colour indicates combination points that were evaluated in *T. cruzi* trypomastigotes, while dark-grey colour indicates higher combinations also assayed in VERO cells. BZL, benznidazole. LTD, loratadine. CPH, cyproheptadine. CFZ, clofazimine. LTD-CPH-CFZ, combination of the three crystal violet analogues as a single drug. 1/1 IC_50_, refers to the sum of each IC_50_, 15 µM + 10 µM + 5 µM = 30 µM. 1/2 IC_50_, 7.5 µM + 5 µM + 2.5 µM = 15 µM. 1/3 IC_50_, 5 µM + 3.33 µM + 1.67 µM = 10 µM. 1/4 IC_50_, 3.75 µM + 2.5 µM + 1.25 µM = 7.5 µM. 1/5 IC_50_, 3 µM + 2 µM + 1 µM = 6 µM.
